# Wound healing mechanism of antimicrobial peptide cathelicidin-DM

**DOI:** 10.3389/fbioe.2022.977159

**Published:** 2022-11-07

**Authors:** Guixi Wang, Zhizhi Chen, Pan Tian, Qinqin Han, Jinyang Zhang, A-Mei Zhang, Yuzhu Song

**Affiliations:** ^1^ Research Center of Molecular Medicine of Yunnan Province, Faculty of Life Science and Technology, Kunming University of Science and Technology, Kunming, China; ^2^ School of Medicine, Kunming University of Science and Technology, Kunming, China

**Keywords:** antimicrobial peptide, cathelicidin-DM, wound healing, skin, infection

## Abstract

**Background and Purpose:** Chronic wound infections and the development of antibiotic resistance are serious clinical problems that affect millions of people worldwide. Cathelicidin-DM, an antimicrobial peptide from *Duttaphrynus melanostictus*, has powerful antimicrobial activity and wound healing efficacy. So, it could be a potential candidate to address this problem. In this paper, we investigate the wound healing mechanism of cathelicidin-DM to establish a basis for preclinical studies of the drug.

**Experimental Approach:** The effects of cathelicidin-DM on cell proliferation and migration, cytokines, and mitogen-activated protein kinase (MAPK) signaling pathways were examined. Then mice whole skin wound model was constructed to evaluate the wound healing activity of cathelicidin-DM, and further histological changes in the wounds were assessed by hematoxylin-eosin staining (H&E) and immunohistochemical assays.

**Key Results:** Cathelicidin-DM promotes the proliferation of HaCaT, HSF, and HUVEC cells in a concentration-dependent manner and the migration of HSF, HUVEC, and RAW.264.7 cells. Moreover,cathelicidin-DM can involve in wound healing through activation of the MAPK signaling pathway by upregulating phosphorylation of ERK, JNK, and P38. However, cathelicidin-DM didn’t affect the secretion of IL-6 and TNF-α. At the animal level, cathelicidin-DM accelerated skin wound healing and early debridement in mice as well as promoted re-epithelialization and granulation tissue formation, α-SMA expression, and collagen I deposition in mice.

**Conclusion and Implications:** Our data suggest that cathelicidin-DM can be engaged in the healing of infected and non-infected wounds through multiple pathways, providing a new strategy for the treatment of infected chronic wounds.

## 1 Introduction

As the first organ of the organism, the skin consists of two parts, the epidermis and the dermis. It is the first barrier against various microbial attacks and plays an important role in protecting the organism and maintaining the homeostasis of the internal environment (Baroni et al., 2012). Trauma inevitably occurred when the skin was exposed to the environment for long periods and was subjected to multiple factors and stresses. Following the onset of skin injury, the four phases of the wound healing processes will be sequentially initiated: hemostasis, inflammation, proliferation, and remodeling, with neighboring phases, linked and overlapping with each other (Wilkinson and Hardman, 2020). To a certain extent, the structural complexity and functional diversity of the skin determine that wound healing is a complex process that involves a variety of cells, cytokines, and various extracellular matrices to complete tissue repair. In the hemostatic phase, this phase is mainly concerned with coagulation and hemostasis, both to prevent massive blood loss that could damage organs and to provide an environment for later cell growth (Wang et al., 2018); in the inflammatory phase, neutrophils and macrophages infiltrate the wound site to engulf and remove cellular debris, bacteria, etc. to prevent bacterial infection (Eming et al., 2007; Tziotzios et al., 2012; Kloc et al., 2019); during the proliferative phase, the activated of keratin forming cells, fibroblasts, and endothelial cells proliferate and migrate together to complete re-epithelialization and granulation of tissue formation (Gonzalez et al., 2016; Wilkinson and Hardman, 2020); during the remodeling phase, collagen Ⅰ is gradually replaced by collagen III and fibroblasts are converted to myofibroblasts, both of which together accomplish the contraction of the wound and scar (Almadani et al., 2021; Mathew-Steiner et al., 2021).

Chronic wounds caused by ubiquitous pathogens are a public safety problem worldwide and would also carry a huge economic burden. Damaged skin loses its barrier function and is at risk of infection (e.g., *Staphylococcus aureus*, *Pseudomonas aeruginosa*, etc.), as well as providing environmental conditions for the growth and colonisation of pathogens, thereby prolonging wound healing and threatening lives (Siddiqui and Bernstein, 2010). The theory that time can heal wounds will not be defeated (Martin, 2020). As drug resistance increases, so will the difficulty of treating such chronic wounds. Some researchers predict that by 2050, 10 million people worldwide will die from drug-resistant microbes (GradisteanuPircalabioru et al., 2021). Traditional therapeutic drugs also do not meet the real needs of society and the clinic and have major shortcomings. The use of intravenous antibiotics can be used to prevent or treat wound infections (Thapa et al., 2020), but this increases the local production of drug-resistant bacteria. In addition, topical antibiotics tend to cause discomfort and contact dermatitis. Therefore, it is necessary to develop novel drugs to manage such wounds.

Antimicrobial peptides are considered to be a new therapeutic strategy for infected non-healing wounds in need of a new treatment (de Souza et al., 2022), as well as an effective alternative to antibiotics (Magana et al., 2020). This is due to their powerful antibacterial and bactericidal activity, low drug resistance and wound healing activity. As a result, an increasing number of researchers are attracted to invest in the development and exploitation of antimicrobial peptides. In recent years, some antimicrobial peptides have also been found to enhance wound recovery (e.g., *Tylotion* (Mu et al., 2014), AH90 (Liu et al., 2014a), CW49(Liu et al., 2014b), Temporins A and B (Di Grazia et al., 2014), Tiger17 (Tang et al., 2014), cathelicidin-OA1 (Cao et al., 2018), cathelicidin-NV (Wu et al., 2018), DRGN-1 (Chung et al., 2017), LL-37 (Saporito et al., 2018; Yang et al., 2020),etc.), which provides better drug candidates for the treatment wounds. They are involved in certain processes of wound healing that play a role in accelerating wound repair which can be broadly summarised as: promotion of cell proliferation and migration, angiogenesis, immune regulation, collagen deposition, conversion of fibroblasts to myofibroblasts, etc (Table 1).

**TABLE 1:** Natural and synthetic antimicrobial peptides in wound healing.

Name	Source	Wound healing features	References
*Tylotoin*	*Salamanders*	Immunomodulatory activity, the ability to promote cell migration and proliferation, and promote angiogenesis	Mu et al. (2014)
AH90	*Odorrana grahami*	Stimulation of TGF-β secretion, Keratinocyte migration, and fibroblast-to-myofibroblast transition	Liu et al. (2014a)
CW49	*Odorrana grahami*	Inhibits excessive inflammation and angiogenesis	Liu et al. (2014b)
Temporins A and B	*Rana temporaria*	Keratinocyte Proliferation and migration, promote angiogenesis	Di Grazia et al. (2014)
Tiger17		Stimulation of TGF-β and IL-6 secretion, Keratinocyte Proliferation and migration, fibroblast-to-myofibroblast transition	Tang et al. (2014)
Cathelicidin-OA1	*Odorrana andersonii*	macrophage recruitment, Keratinocyte proliferation and Fibroblast migration	Cao et al. (2018)
Cathelicidin-Nv	frog *Nanorana ventripunctata*	Keratinocyte and Fibroblast Proliferation, fibroblast-to-myofibroblast transition, collagen production in fibroblasts	Wu et al. (2018)
DRGN-1	*Varanus komodoensis*	granulation tissue formation, re-epithelialization, and keratinocyte proliferation/migration	Chung et al. (2017)
LL-37	human	Induction of cell proliferation, migration, and angiogenesis	Saporito et al. (2018), Yang et al. (2020)

Cathelicidin DM is a bifunctional peptide (Shi et al., 2020). It kills a wide range of bacteria, and even inhibits clinical isolates. On the other hand, in a wound model of *E. coli* infection, the wound healing rate in mice treated with cathelicidin-DM was superior to that in the control and gentamicin groups. This paper aims to investigate the wound healing mechanism of cathelicidin-DM based on the wound healing process. It could provide preclinical data for its development as a drug for the prevention or treatment of wound infection and wound healing, or even for the treatment of chronic wounds with infection.

## 2 Material and methods

### 2.1 Material

Dulbecco’s modified eagle medium (DMEM) and fetal bovine serum (FBS) respectively purchased from gibco in the US and Biological Industries in Israel. MAPK Family Antibodies Sampler Kit (Cat# 9926, RRID:AB_330797) and Phospho-MAPK Family Antibodies Sampler Kit (Cat# 9910, RRID:AB_330792) provided by Cell Signaling Technology. 4% paraformaldehyde, paraffin, phenylmethylsulfonyl fluoride, protein phosphatase inhibitors, etc., were purchased from China Soleibao Company.

Balb/c mice were purchased from Kunming Medical University. All cells were obtained from Kunming Institute of Zoology, Chinese Academy of Sciences. It was approved by the Experimental Animal Ethics Committee of Kunming University of Science and Technology for the work to be carried out.

### 2.2 Synthesis of cathelicidin-DM

Cathelicidin-DM, synthesized by Hangzhou DGpeptides, has been determined to have a molecular mass of 4,163.97 and a purity of >95% after Mass Spectrometry and High Performance Liquid Chromatography analysis.

### 2.3 Analysis of cell proliferation assay

The effect of cathelicidin-DM on the proliferation of human umbilical vein endothelial cells (HUVEC, RRID:CVCL_2959), human immortalized keratinocytes (HaCaT, RRID:CVCL_0038), and human skin fibroblasts (HSF, RRID:CVCL_9V78) were cultured with DMEM and used the CCK-8 assay. The concentrations of trypsin-digested resuspended cells were counted separately using hemocytometer plates and inoculated in 96-well plates (5 × 10^3^ cells/well, 90 μl). After the cells were plastered, 10 μl of cathelicidin-DM was added at final concentrations of 0, 2, 5, 10, and 20 μg/ml and incubated in a 5% CO_2_ incubator at 37°C for 16 h. Add 10 μl of CCK-8 to each well to continue the incubation for 1 h, keeping in mind that this step requires protection from light. The absorbance is measured at 450 nm, which reflects the number of cells.

### 2.4 Cell migration assay

The influence of cathelicidin-DM on the migration of HUVEC and HSF was examined using a cell scratch assay (Mu et al., 2014). The digested and counted HUVEC and HSF cells were spread out on the plate and left to culture until the cells reached about 90% melting. The serum-free DMEM was then replaced for cell starvation. After leaving for 24 h, the plates were scored with a 200 μl gun tip. After scoring, the plates were washed 3 times with PBS to remove the scoring cells and the serum-free medium was added for culture. Cathelicidin-DM at a final concentration of 20 μg/ml was also added and the control group was added to the serum-free medium, and the migration of cells was photographed and recorded at 0, 12, 24 and 48 h respectively. ImageJ and Photoshop were used to process the change in the scratch area and mark the location of cell migration respectively. Photographs were taken at the same location to ensure the reliability of the experiment.

### 2.5 Effects on macrophages *in vitro*

To further validate the biological function of cathelicidin-DM on macrophages, We used a transwell migration assay to examine the effect of cathelicidin-DM on the migration of mouse RAW264.7 cells (RRID:CVCL_0493) (Mi et al., 2018). The cells were starved for 10 h before preparing the cell suspension to reduce the effect of serum. Next, the chambers were equilibrated for 2 h using 200 μl of serum-free DMEM to hydrate the basement membrane. The cells were digested, counted, and adjusted to a cell concentration of 1.0 × 10^5^ cells/ml. 200 μl of the cell suspension with PBS or cathelicidin-DM was added to the upper chamber of the transwell, while the lower chamber was added to a medium containing 20% FBS and incubated in a cell culture incubator. After 24 h of incubation, the transwell chambers were removed, washed with PBS (2 times), fixed with 4% paraformaldehyde for 15 min, washed with PBS 2 times, stained with 0.5% crystal violet (10 min), and washed with PBS 3 times, and the transwell chambers were placed upside down on filter paper until they were air-dried and photographed under an inverted microscope.

### 2.6 Cytokine detection

RAW264.7 cells were digested at the logarithmic growth stage and grown in 96 well plates (180 μl, 1 × 10^5^ cells/well). Then, the cells were treated with different final concentrations of cathelicidin-DM for 24 h. The supernatant was collected and the effect of cathelicidin-DM on the secretion of IL-6 and TNF-α was measured using an enzyme linked immunosorbent assay (ELISA) kit, refer to the instructions for details.

### 2.7 Mitogen-activated protein kinase signaling pathway assay

Cultured mouse RAW264.7 cells were digested, counted, inoculated into 6-well culture plates (2 × 10^6^ cells/well), placed in a 5% CO_2_ incubator at 37°C, and allowed to grow to 80% fusion, then transferred to serum-free DMEM and starved for 16 h. The cells were treated with different final concentrations (0, 2, 5, 10, and 20 μg/ml) of cathelicidin-DM for 3 h, then washed twice with pre-cooled PBS solution, and incubated for 30 min on ice with 250 μl of High-Performance RIPA Lysis Buffer containing 1% PMSF. Phosphorylated proteins also require the addition of dephosphorylation inhibitors. The lysate is scraped off using a cell scraper and transferred to a 1.5 ml centrifuge tube at 4°C for 20 min at 12000 rpm. The supernatant is transferred to a new 1.5 ml tube and the supernatant is the total protein. The supernatant was transferred to a new 1.5 ml centrifuge tube. The concentration of protein was determined using the BCA kit, refer to the instructions. The remaining proteins were separated and stored at −20°C.

The protein samples added with 5x loading buffer were placed in a 98°C metal bath and boiled for 10 min. The denatured protein samples were separated by 12% SDS-PAGE gel electrophoresis, and the separated proteins were transferred to a polyvinylidene fluoride membrane at the same time. PVDF was blocked with 5% BCA for 2 h at room temperature. After blocking, the membrane containing the target protein was washed three times with PBST buffer for 15 min each time. Select the corresponding primary antibody according to the desired target protein, and dilute the antibody with PBST at a ratio of 1:2000. The primary antibody was added to the incubation box and incubated overnight at 4°C. The next morning, the membrane was washed five times with PBST solution for 5 min each time, and then HRP-labeled goat anti-rabbit IgG (1:1000) was added, and incubated at 37°C for 1 h. The membrane was washed 5 times with PBST solution, 5 min each time, and developed with a developing instrument.

### 2.8 Construction of model of whole-layer trauma in mice

When the mice were purchased, they were kept in separate cages in the laboratory for 1 week to adapt to the environment and to ensure the reliability of the experiment. We randomly divided the mice into the control group and the experimental group (*n* = 5). The mice were anesthetized by intraperitoneal injection of sodium pentobarbital, removed their hair, and disinfected skin with 75% medical alcohol. A full skin trauma model of approximately 6 mm in diameter was made on the back of mice (He et al., 2019).

### 2.8.1 Construction of a non-infected model of whole-layer trauma in mice

The control and experimental groups were respectively treated with a sterile PBS solution and 300 μg/ml cathelicidin-DM at 12-h intervals. Photographs were taken of the mice on days 0, 2, 4, 6, 8, 10, and 12 days to record wound healing. The area was calculated using ImageJ software. Photographs were taken at the same location and at the same level to ensure the accuracy of the experiment.

### 2.8.2 Construction of an infection model for whole-layer trauma in mice

*S. aureus* (ATCC 25923) was used to infect mouse trauma sites to produce a mouse whole-layer trauma infection model. *Staphylococcus aureus* was first resuscitated by manipulation in a biological ultra-clean table. A single clone was picked and incubated in LB medium at 37°C and 200 rpm. When the logarithmic stage of growth was reached, the concentration of the bacterial solution was measured using an Ultraviolet-visible Spectrophotometer (1OD = 1 × 10^9^ cells/ml). The above *S. aureus* solution was adjusted to a concentration of 1 × 10^8^ cells/ml to infect the wounds of mice. After *S. aureus* infection, the subsequent steps are the same as in 2.8.1.

### 2.9 Hematoxylin-eosin staining staining and immunohistochemical analysis

The method was modified slightly according to 2.8.1. Symmetrical full-thickness skin wounds of 6 mm were constructed on both sides of the back of the same mouse and treated with PBS and cathelicidin DM respectively. Wound tissue with a small amount of normal tissue was taken at the appropriate time and preserved in 4% paraformaldehyde solution for H&E staining and immunohistochemistry referring to the literature or instructions for the exact procedure. Granulation tissue and epidermis in H&E stained sections were assessed using a semi-quantitative scoring system (Galeano et al., 2003; Liu et al., 2014a). The system uses a four-point scoring method to evaluate the formation of granulation tissue. one to four points represent a thin granulation layer, moderate granulation layer, thick granulation layer, and very thick granulation layer respectively. skin dermal and epidermal regeneration was evaluated by three-point scoring (1, little regeneration; 2, moderate regeneration; and 3, complete regeneration). Importantly, dermis and epidermis regeneration, and granulation tissue are quantified using Image Pro Plus. The immunohistochemical section was analyzed by ImageJ.

### 2.10 Statistical analysis

All data were analyzed using Student’s t-tests or one-way ANOVA provided by GraphPad prism 8. Experimental results were expressed as mean ± standard deviation. *p* < 0.05 was considered statistically significant between the two groups.

## 3 Results

### 3.1 Cathelicidin-DM promoted the proliferation of HaCaT, HSF, and HUVEC cells

**FIGURE 1:**
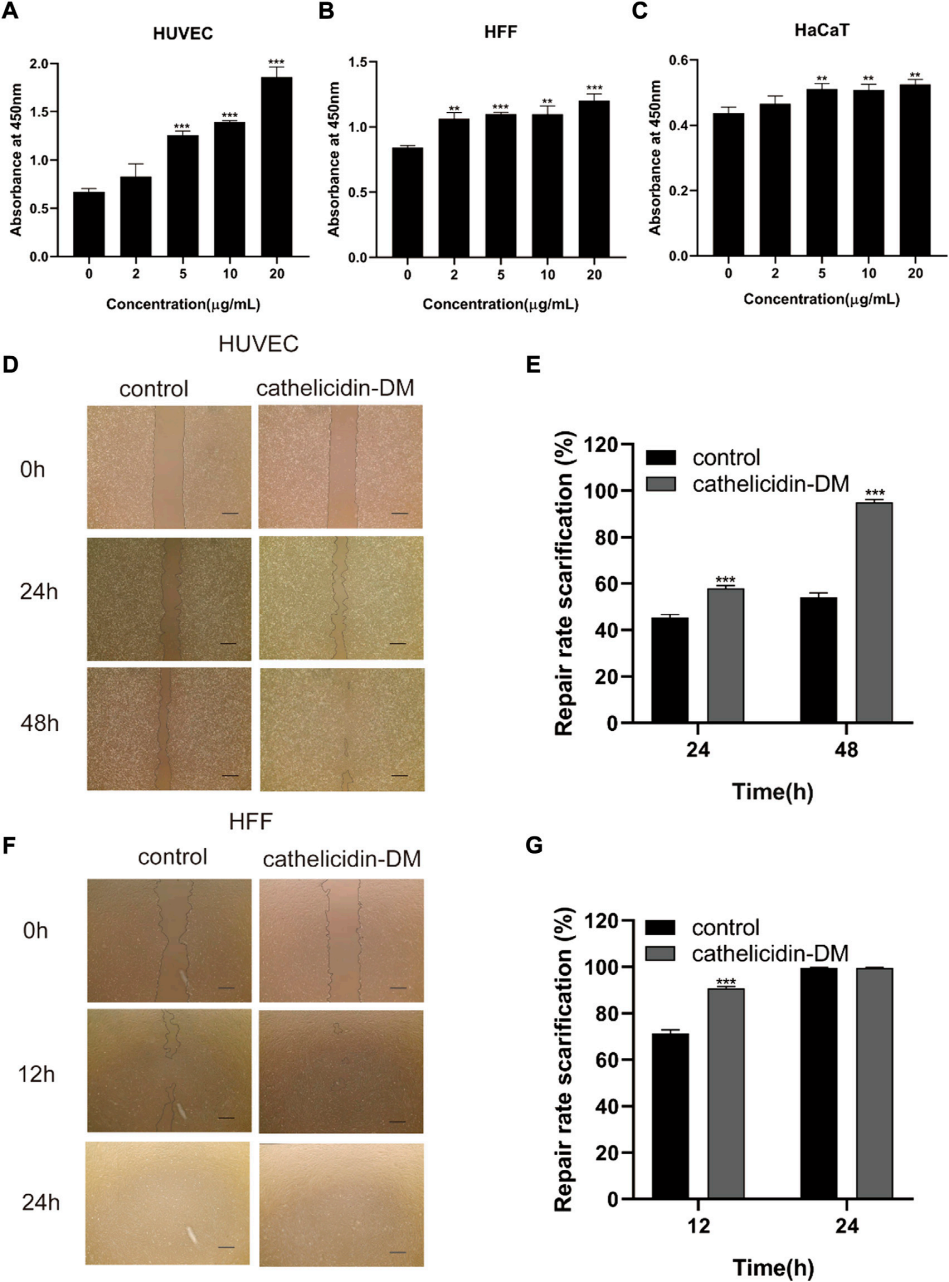
Effect of cathelicidin-DM on cell proliferation and migration. **(A-C)** The influence of different concentrations of cathelicidin-DM on the viability of HUVEC, HFF, and HaCaT cells at 0, 2, 5, 10, and 20 μg/ml, respectively. **(D,F)** Migration of HUVEC and HFF cells stimulated by 20 μg/ml cathelicidin-DM, Scale bar represents 200 μm. **(E,G)** The microscope images were quantified for analysis using ImageJ software and cell migration rates were calculated in the area of the cell scratches. All the experiments were repeated 3 times and the data are expressed as mean ± standard, *p < 0.05, **p < 0.01, ***p < 0.001.

The skin is an organ based on keratinocytes, fibroblasts, vascular endothelial cells, and other cells whose proliferative and migratory activities are particularly important for wound repair. Studies have indicated that antimicrobial peptides can induce cell proliferation and migration to accelerate tissue healing, such as Cathelicidin-NV (Wu et al., 2018), SR-0379 (Tomioka et al., 2014), DRGN-1 (Chung et al., 2017), LL-37 (Koczulla et al., 2003; Carretero et al., 2008), etc. Therefore, we investigated the effect of different concentrations of cathelicidin-DM on HUVEC, HSF, and HaCaT cell viability using the CCK-8 assay. The results showed that cathelicidin-DM accelerated the proliferation of HUVEC, HSF, and HaCaT cells in a dose-dependent manner. As shown in Figures 1A–C, compared with the control group, the growth rates of HaCaT, HSF, and HUVEC cells were 16.27% and 19.92%, 30.5% and 42.58%, 107.32% and 177.17% at cathelicidin-DM concentrations of 10 μg/ml and 20 μg/ml, respectively.

### 3.2 Cathelicidin-DM facilitates the migration of HUVEC and HSF cells

Since cathelicidin-DM was more powerful in promoting the growth of HSF and HUVEC cells, we investigated the effect of cathelicidin-DM on the migration of HUVEC and HSF cells using a cell scratch assay. It was revealed that cathelicidin-DM significantly enhanced the migration of HUVEC (Figures 1D–G), while the effect on the migration of HSF cells was greater before 12 h and decreased with time. Cathelicidin-DM had 58.0% and 95.1% scratch repair rates for HUVEC cells at 24 and 48 h, whereas the control group only had 45.5% and 54.3%; the scratch repair rate for HSF cells treated with cathelicidin-DM was 90.4% and 99.6% at 12 and 24 h, while the scratch repair rate for the control group was 71.5% and 99.6%.

### 3.3 Cathelicidin-DM induced macrophage recruitment

**FIGURE 2:**
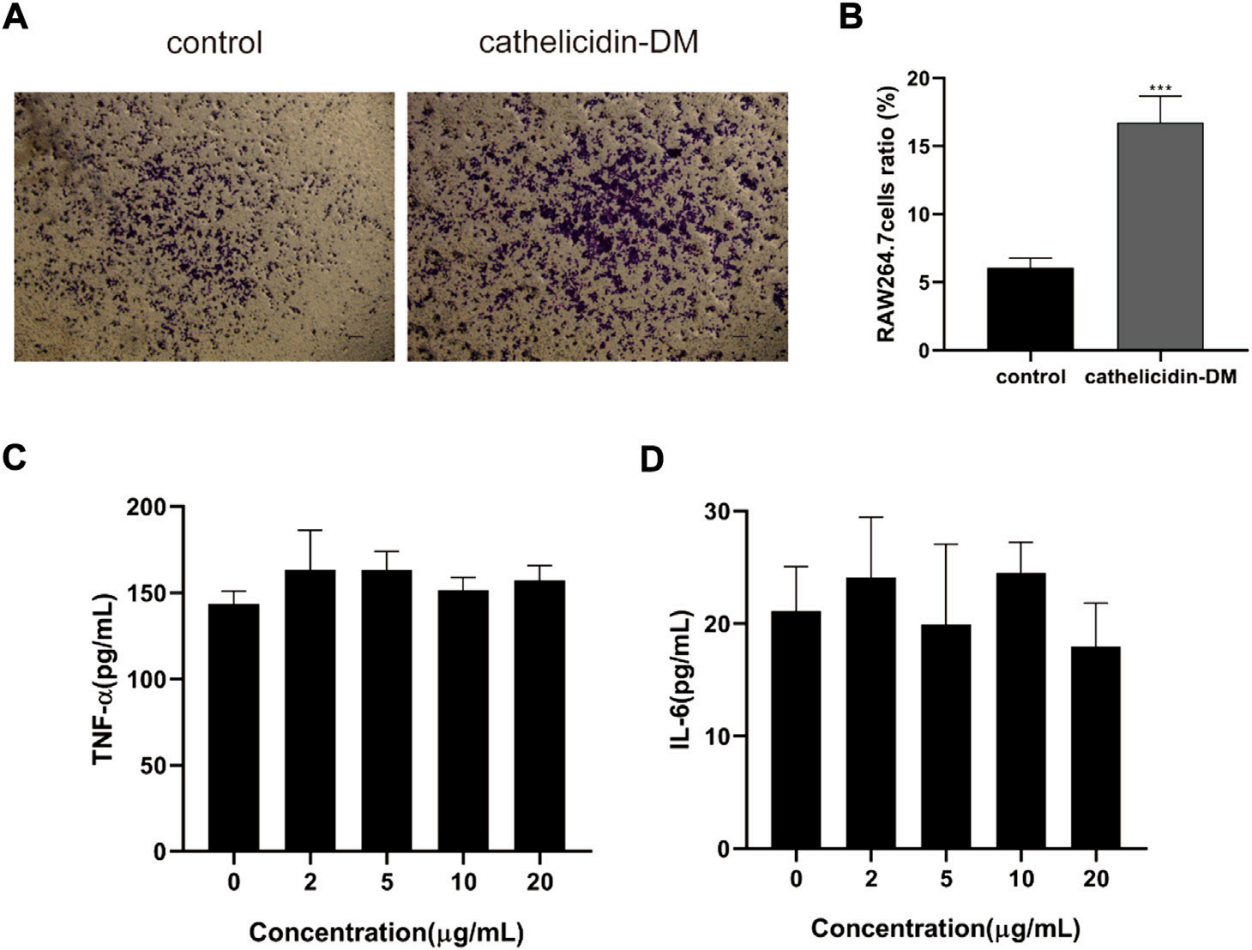
Effect of cathelicidin-DM on the migration of mouse RAW264.7 cells and secretion of TNF-α and IL-6. **(A)** The effect of 20 μg/ml cathelicidin-DM on the migratory activity of mouse RAW264.7 cells was assayed using Transwell chambers, Scale bar represents 100 μm. **(B)** Quantitative analysis of cells in **(A)** with ImageJ. **(C,D)** The effect of different concentrations of cathelicidin-DM on the secretion of TNF-α, IL-6. All the experiments were repeated 3 times and the data are expressed as mean ± standard, *p < 0.05, **p < 0.01, ***p < 0.001.

Macrophages are involved in the entire process of wound healing, especially during the inflammatory phase, and also produce chemokines and growth factors such as TNF-α, IL-6 TGF-β1, and VEGF-α (Mi et al., 2018; Kloc et al., 2019), which promote cell proliferation and migration, and promote angiogenesis. Tiger17 from frogs and tylotoin from salamanders are chemotactic and recruit macrophages to the wound site to remove damaged tissue and antigens (Mu et al., 2014; Tang et al., 2014). As displayed in Figures 2A,B, cathelicidin-DM was able to promote the migration of mouse RAW264.7 cells *in vitro*, which improved cell migration performance by approximately 2.6-fold compared to the control group. Further, we examined the effect of cathelicidin-DM on the secretion of TNF-α and IL-6 by RAW264.7 cells using ELISA. As shown in Figure 2, 5, the results indicate that cathelicidin-DM had no significant effect on the secretion of TNF-α and IL-6 compared to the control group. (Figures 2C,D).

### 3.4 Cathelicidin-DM activates mitogen-activated protein kinase signaling pathway for injury healing

**FIGURE 3:**
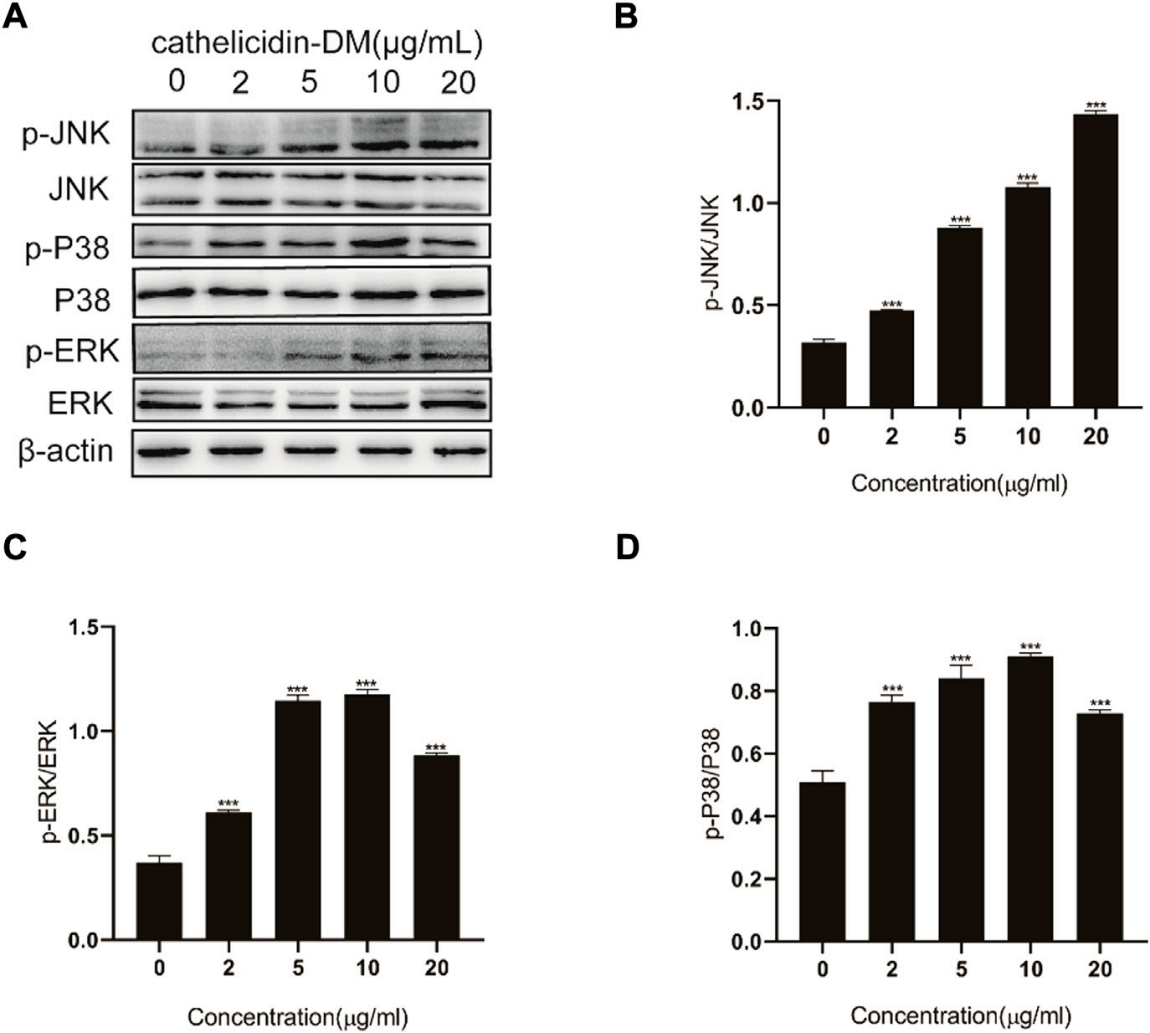
Effect of cathelicidin-DM on MAPK signaling pathway. **(A)** Western blot demonstrates the effect of cathelicidin-DM on the phosphorylation of JNK, ERK, and P38. **(B–D)** Relative greyscale analysis by ImageJ and relative activation by cathelicidin-DM of JNK **(B)**, ERK **(C)**, and P38 **(D)** expression. Values for the cathelicidin-DM treated group were significantly different from control groups. *p < 0.05, **p < 0.01 (*n* = 3).

Since the MAPK signaling pathway has a role in wound healing and is closely related to cell proliferation, differentiation, and migration (Wu et al., 2018), we speculate that cathelicidin-DM activates the MAPK signaling pathway when it exerts its wound-healing function. As indicated in Figure 3A, we observed inducable extracellular regulated protein kinases (ERK), c-Jun N-terminal kinase (JNK), and P38 Mitogen-Activated Protein Kinase (P38) phosphorylation using western blot. Cathelicidin-DM regulated JNK phosphorylation in a concentration-dependent manner, and no concentration-dependent regulation was shown for ERK and P38, which were phosphorylated to the highest extent at a cathelicidin-DM concentration of 10 μg/ml (Figures 3B–D). In comparison with the control group, the phosphorylation levels of ERK, P38, and JNK were respectively up-regulated by 139.2%, 43.0%, and 348% at a cathelicidin-DM concentration of 20 μg/ml. In conclusion, cathelicidin-DM activates phosphorylation of ERK, JNK, and P38 to activate MAPK signaling pathway to contribute to skin wound healing.

### 3.5 Cathelicidin-DM accelerated whole skin wound healing in mice

**FIGURE 4:**
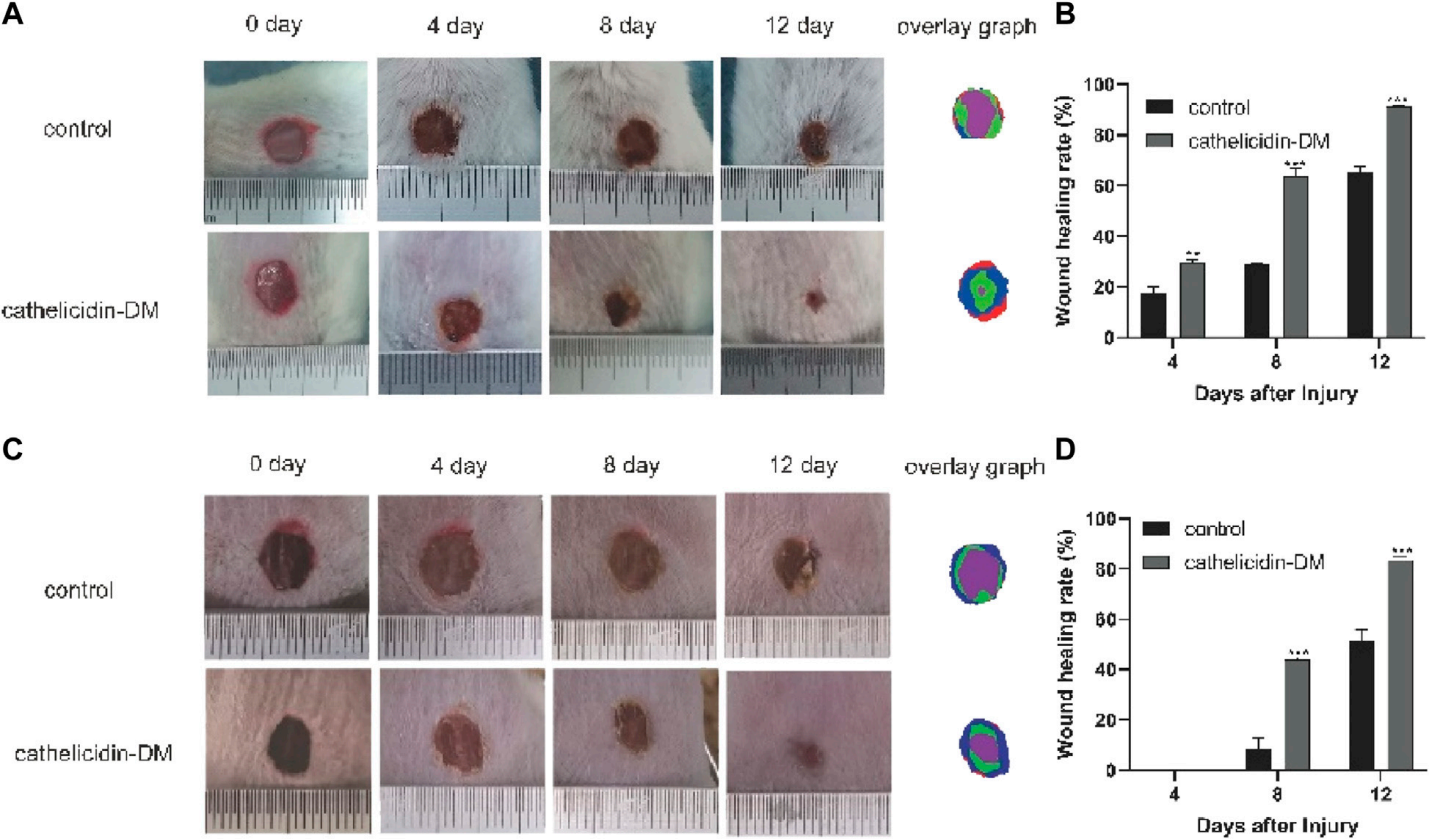
Cathelicidin-DM treatment accelerates wound closure in balb/c mice. **(A)** Graph of the healing effect of mouse trauma models treated with cathelicidin-DM, PBS on days 0, 4, 8, and 12 after trauma and superimposed maps of wound tissues from mice at 0, 4, 8, and 12 d processed by Photoshop, with red, blue, green and purple representing wound tissues from mice at 0, 4, 8 and 12 d post-trauma, correspondingly. **(B,D)** Quantitative analysis of wounds using ImageJ software and calculation of wound healing rates in the control and cathelicidin-DM groups. **(C)** Healing effects and wound overlays of a mouse S. aureus-infected wound model treated with cathelicidin-DM, PBS on days 0, 4, 8, and 12 post-wound. The value is shown as mean ± standard, *p < 0.05, **p < 0.01.

Previous work had demonstrated that caudal intravenous injection of cathelicidin-DM could therapeutic healing of *E. coli* infected wounds (Shi et al., 2020). And cathelicidin-DM could facilitate cell proliferation and migration. So we constructed a full-skin non-infected wound mice model to estimate the wound healing activity of topical cathelicidin-DM, observed the mice daily and photographed changes in wound area in mice. Figure 4A shows wound healing in mice at 0, 4, 8, and 12 days postoperatively, indicating that mice treated with topical cathelicidin-DM exhibited significant wound healing, much faster than the control group. We then analyzed the wound healing rates of the control and cathelicidin-DM treated mice and superimposed the wound tissue at different times, as shown in Figures 4B,C. It is clear that the wounds of the cathelicidin-DM treated mice were almost completely healed at 12 d. The results showed that the wound healing rate of mice treated with topical cathelicidin-DM reached 30%, 65%, and 91% at 4, 8, and 12 days post-trauma, respectively, compared to only 21%, 28%, and 66% in the control group, which indicates that the wounds of cathelicidin-DM treated mice were almost completely healed at 12 days. In another experiment, we found that topical application of cathelicidin-DM accelerated skin wound healing in *S. aureus*-infected mice compared with the control group (Figures 4C,D). 83.34% wound healing was achieved in the cathelicidin-DM-treated group at 12 d. In conclusion, cathelicidin-DM exerted therapeutic effects on both non-infected and S. aureus-infected wounds.

### 3.6 Hematoxylin-eosin staining dyeing analysis

**FIGURE 5:**
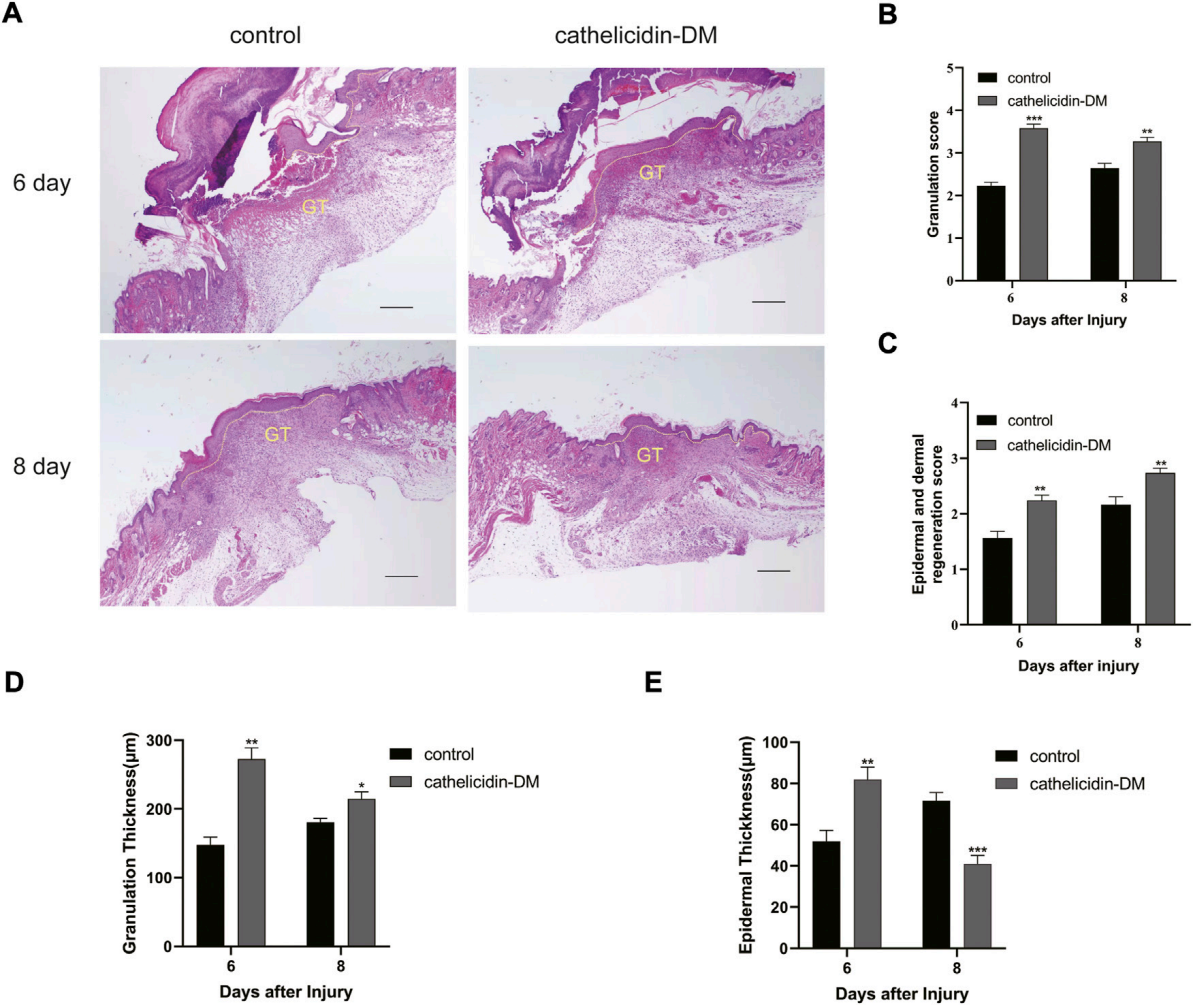
Histopathological examination of wounds in mice. **(A)** Effect of cathelicidin-DM on skin re-epithelialization and granulation tissue in mice (note: dashed line represents reepithelialization, GT represents granulation tissue, bars indicate 200 μm). **(B–E)** Histological scores of granulation thickness, epidermis and dermis regeneration; granulation and epidermal thickness of mice. Values represent means ± standard (*n* = 3). *p < 0.05, **p < 0.01.

Tissue re-epithelialization and granulation tissue formation are crucial aspects of the proliferative phase of wound healing. As shown in Figure 5, cathelicidin-DM promoted tissue re-epithelialization and granulation tissue formation in mice, while the wound length was less than that treated with PBS. On day 6 post-trauma, the cathelicidin-DM group had a thicker epidermis and more abundant granulation tissue compared to the blank group. On the 8th day after wounding, mice in the cathelicidin-DM group still had more wound granulation tissue than the group treated with PBS, and the epidermis was thinner and changed to normal tissue.

### 3.7 Cathelicidin-DM facilitated the expression of α-smooth muscle actin and increased the deposition of collagen I

α-SMA is a symbol of differentiation of fibroblasts into myofibroblasts, which can traction wound contraction (Pfalzgraff et al., 2018). In previous studies, AH90 and tylotion exert wound healing effects through the conversion of fibroblasts to myofibroblasts, while Epinecidin-1 takes effect by increasing collagen formation (Liu et al., 2014a; Mu et al., 2014; Huang et al., 2017).

We extracted wound tissue from mice at 6 and 8 days post-trauma and further explored by immunohistochemistry whether cathelicidin-DM affects wound healing activity through α-SMA expression and collagen I deposition during the remodeling phase. The results showed that cathelicidin-DM promoted the expression of α-SMA and increased the deposition of collagen I (Figure 5). We found that the ratio of α-SMA positive area was 18% and 26.5% in the cathelicidin-DM treated group, respectively, compared to only 5.1% and 12.0% in the PBS group. The collagen-positive area was 3.6 and 2.2 times higher in the cathelicidin-DM group than in the control group at 6 and 8 d post-trauma respectively (Figures 5B,D).

## 4 Discussion

Cathelicidin-DM provides a better drug candidate molecule for the treatment of chronic wound infections (Shi et al., 2020). It shows strong antibacterial ability, which the MIC of cathelicidin-DM is as low as 6 μg/ml. This involves MDR and XDR, such as *Staphylococcus haemolyticus* (CI 1541410970016), *Enterococcus faecalis* (MDR 14U0445), *Staphylococcus aureus* (ATCC25923), *Escherichia coli* (MDR 13A10022), *K Pneumonia* (XDR 13A13361), etc. Antibacterial mechanism of cathelicidin-DM was confirmed to be related to the membrane permeability in the SYTOX green absorption experiment.

It also has the advantage of wound healing activity in the wound model of *E. coli* infection… *Staphylococcus aureus*, one of the common pathogens of community and hospital infection, widely exists in the natural environment and can cause a variety of serious infections. In addition, cathelicidin DM can treat wounds infected by *Staphylococcus aureus* (Figure 4).

Chronic wounds caused by pathogenic bacteria have always been a difficult area of medical treatment. Currently, wound medication is mainly used to prevent and treat wound infections with antibiotics, whose use is limited due to the rapid development of drug resistance. Some bifunctional peptides with antimicrobial and wound healing activities have great potential for the prevention and treatment of infectious wounds (Miao et al., 2021). Therefore, the wound-healing mechanism of cathelicidin-DM was investigated.

Cutaneous wound repair is a complex, conservative physiological process comprising four successive and overlapping phases of hemostasis, inflammation, proliferation, and remodeling, which work in harmony with each other to complete the repair of tissue and restore normal function (Barrientos et al., 2008; Sorg et al., 2017). The wound healing process is a cell-based repair process in which each cell performs different roles in the wound healing process. Macrophages participate in the entire phase of wound healing, especially during the inflammatory phase (Kim and Nair, 2019). During this phase, macrophages can be recruited to the wound and engulf apoptotic or dead cells, microorganisms, etc. which can be differentiated into M1 and M2 type macrophages to exert anti-inflammatory and pro-inflammatory effects (Kloc et al., 2019). *Tylotoin*, cathelicidin-OA1, AH90, and Tiger1 may facilitate macrophage recruitment or the release of factors involved in the inflammatory phase of wound healing. *In vitro*, experiments have shown that cathelicidin-DM promotes the migration of RAW264.7 cells, but does not affect the secretion of IL-6 and TNF-α (Figure 2).

During the proliferative phase of wound healing, keratinocytes are the structural cells of the healing process, in case of skin injury, keratin-forming cells at the wound margin receive signals and promote proliferation and migration for re-epithelialization of the tissue (Sorg et al., 2017). Fibroblasts and vascular endothelial cells are a necessary part of the granulation tissue that fills the injured area. Vascular endothelial cells, which are stimulated by several factors to migrate and form capillaries, are important conduits for the transport of nutrients, oxygen, and other substances (Dulmovits and Herman, 2012; Sorg et al., 2018). Most wound healing peptides can be demonstrated, e.g., Temporins A, Tylotoin, Cathelicidin-OA1. Cathelicidin-DM facilitated the proliferation of HUVEC, HSF, and HaCaT cells and the migration of HUVEC and HSF cells (Figure 1). This in turn accelerated re-epithelialization and granulation tissue formation at the site of skin injury in mice. (Figure 5). Western blot showed that cathelicidin-DM upregulated the phosphorylation of JNK, ERK, and P38 in the MAPK signaling pathway to activate the MAPK signaling pathway in wound healing, which is involved in cell proliferation, and differentiation (Figure 3).

**FIGURE 6:**
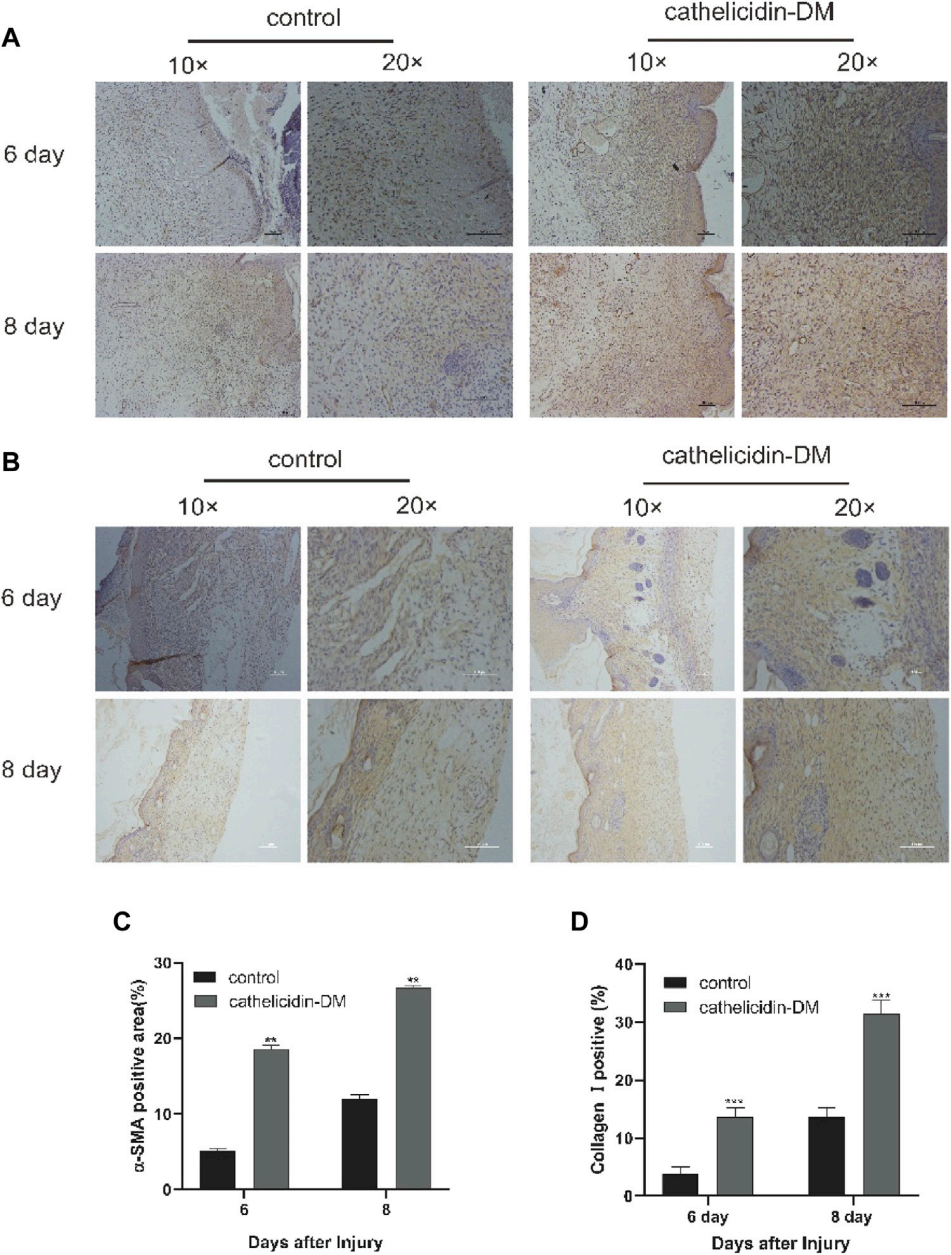
Cathelicidin-DM facilitated the expression of α-SMA and increased the deposition of collagen I. **(A)** Immunohistochemical picture of α-SMA-labelled skin tissue sections at 6 and 8 d post-trauma, with α-SMA staining in brown and scale representing 200 μm. **(B)** Images of wound skin sections stained with anti-collagen I at 6 and 8 d post-trauma, and stained brown with collagen I and scale representing 200 μm. **(C,D)** Positive rates for α-SMA **(C)** and collagen I **(D)** labeling immunohistochemistry were analyzed by ImageJ software. All data are presented as mean ± standard deviation compared to the control group, *p < 0.05, **p < 0.01 (*n* = 3).

Two key features of the remodeling phase of wound healing are the differentiation of fibroblasts into myofibroblasts, which are responsible for wound contraction, and the conversion of collagen III into collagen I and its deposition (Broughton et al., 2006; Pazyar et al., 2014). To this end, cathelicidin-DM was examined to study if it plays a role in the remodeling phase. Immunohistochemical experiments showed that cathelicidin-DM stimulates the expression of α-SMA and the deposition of collagen I. The amount of α-SMA expression indicates the number of myofibroblasts (Figure 6). It suggests that cathelicidin-DM is capable of promoting the differentiation of fibroblasts into myofibroblasts, which may play a traction role in wound contraction.

AMPs application is also challenging in terms of inherent limitations. Current research on AMPs has focused on the identification of potent and selective peptides, as well as mechanisms and modes of action. The researchers found that they showed low stability and bioavailability when facing the local wound environment. In the future we will work in the following directions: 1. Direct targets of cathelicidin-DM and the relationship between structure and function; 2. Enhancement of cathelicidin-DM activity and stability by modifying and modifying peptides.

In summary, cathelicidin-DM is an antimicrobial-wound healing peptide that treats the healing of infected and non-infected wounds through multiple mechanisms. As such, it is expected to be developed as a wound-healing drug to be developed for the prevention or treatment of wound healing in infected skin wounds, offering a new strategy for the treatment of infected chronic wounds.

## Data Availability

The original contributions presented in the study are included in the article/Supplementary Material, further inquiries can be directed to the corresponding author.
