# Supplementary material for: Wound healing mechanism of antimicrobial peptide cathelicidin-DM
Source: Front Bioeng Biotechnol. 2022 Nov 7;10:977159. doi: 10.3389/fbioe.2022.977159 (PMC9681526; doi:10.3389/fbioe.2022.977159)
Supplement: Supplementary file 1 [file DataSheet1.pdf]

Supplementary Material

1 Supplementary Figures

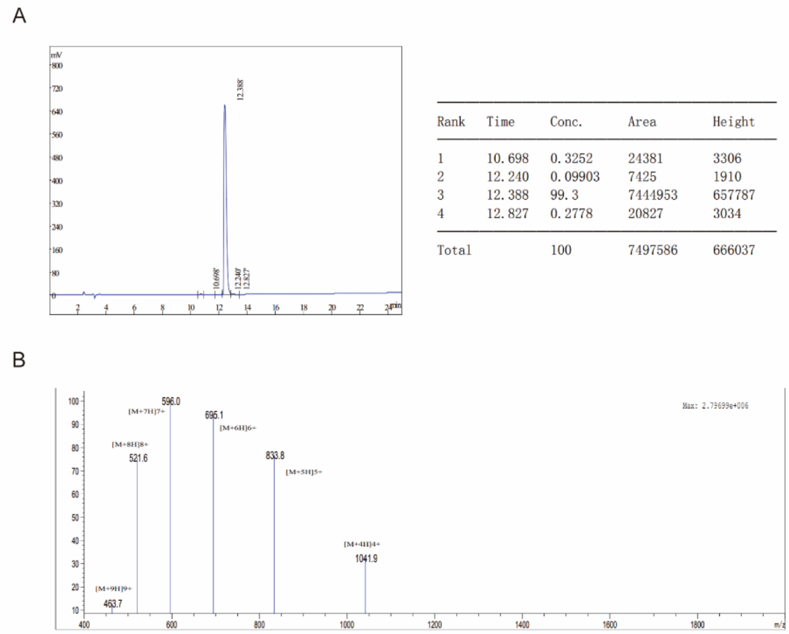

Rank	Time	Conc.	Area	Height
1	10.698	0.3252	24381	3306
2	12.240	0.09903	7425	1910
3	12.388	99.3	7444953	657787
4	12.827	0.2778	20827	3034
Total		100	7497586	666037

Supplementary Figure 1. High performance liquid chromatogram (HPLC) and mass spectra (MS) of cathelicidin-DM. (A) HPLC, retention time and peak area information of synthetic polypeptide cathelicidin-DM, the purity of cathelicidin-DM was >95%; (B) The molecular mass of cathelicidin-DM is 4163.97.
